# Effect of Hyperglycemia at Presentation on Outcomes in Acute Large Artery Occlusion Patients Treated With Solitaire Stent Thrombectomy

**DOI:** 10.3389/fneur.2019.00071

**Published:** 2019-02-19

**Authors:** Xiaochuan Huo, Raynald Liu, Feng Gao, Ning Ma, Dapeng Mo, Xiaoling Liao, Chunjuan Wang, Xuan Sun, Ligang Song, Baixue Jia, Lian Liu, Bo Wang, Yuesong Pan, Yilong Wang, Liping Liu, Xingquan Zhao, Yongjun Wang, Zhongrong Miao, The EAST Study Group

**Affiliations:** ^1^Interventional Neuroradiology Center, Beijing Tiantan Hospital, Capital Medical University, Beijing, China; ^2^Department of Neurology, Beijing Tiantan Hospital, Capital Medical University, Beijing, China

**Keywords:** mechanical thrombectomy, hyperglycemia, acute ischemic stroke, stent retriever, outcomes

## Abstract

**Background:** Sporadic data showed hyperglycemia at presentation is associated with poor outcomes in patients with acute ischemic stroke (AIS) under mechanical thrombectomy (MT) treatment.

**Objective:** This study aims to evaluate the relationship of admission hyperglycemia and outcomes in patients treated with solitaire stent thrombectomy.

**Methods:** This multicenter prospective study registered patients with AIS due to anterior circulation large vessel occlusion (LVO) suitable for MT with Solitaire stent retriever. We analyzed the influence of admission hyperglycemia (≥7.8 mmol/L) and serum glucose on functional independence which is defined as modified Rankin Scale score (mRS) of 0–2, symptomatic intracranial hemorrhage (sICH) and several outcomes of interest using univariable and multiple logistic regression analysis.

**Results:** This study involved 17 stroke centers across China and consecutively recruited 149 patients. Patients with hyperglycemia at presentation less frequently exhibited a functional independence at 3 months than patients without hyperglycemia (22.2 vs. 66.4%; odds ratio 0.75, 95% confidence interval 0.61–0.92; *P* = 0.005). Higher glucose levels were correlated with worse outcome (per 1 mmol/L increase in glucose: odds ratio for mRS score 0–2 at 3 months 0.17, 95% confidence interval 0.06–0.45; *P* < 0.001) at 3 months and sICH (per 1 mmol/L increase in glucose: odds ratio for sICH was 8.2, 95% confidence interval 1.13–29.57; *P* < 0.001) after thrombectomy.

**Conclusions:** Higher admission serum glucose and hyperglycemia were independently correlated with lower functional independence at 3 months in patients treated with Solitaire stent thrombectomy of anterior circulation LVO. Higher admission serum glucose was also associated with sICH after thrombectomy.

## Introduction

The role of glucose in ischemic injury is complex and several studies have shown how hyperglycemia aggravates ischemic brain injury in AIS patients treated with tissue plasminogen activator (tPA) (1–7). After the publication of five positive randomized trials for mechanical thrombectomy (MT), this technique is widely used for large artery occlusion and reperfusion of occluded artery is frequently achieved by stent retriever (8–12). Hyperglycemia has been shown to enhance intracellular acidosis in ischemic penumbra and exacerbate brain injury (4). The expansion in the use of MT may expose more AIS patients with LVO to reperfusion injury and the influence of hyperglycemia may be more obvious in AIS patients after MT reperfusion. However, sporadic data showed hyperglycemia at presentation is associated with poor outcomes in AIS patients receiving MT.

Kim et al. study showed hyperglycemia was correlated with worse outcome at 90 days (13). The harmful effects of hyperglycemia may have been enlarged for incomplete reperfusion patients after endovascular treatment. Furthermore, Goyal et al. research found higher serum glucose at admission could predict an adverse outcome in patients with LVO treated under MT (14). However, this study didn't show effect of hyperglycemia on successful reperfusion patients' clinical outcome after MT and there was no specific study to investigate the role of hyperglycemia in a trial with more intracranial large artery occlusion patients with underlying atherosclerosis that need rescue therapy during MT.

In our study, we aim to evaluate the association of hyperglycemia and clinical outcomes including 90 day's functional outcome, mortality, and sICH in patients who were enrolled in the “Endovascular treatment of acute ischemic stroke (EAST)” study which had more atherosclerosis patients under rescue therapy (15). Underlying intracranial atherosclerosis (ICAS) was identified in about one-third of the patients in EAST study and the “rescue therapy” strategy was to detach the stent retriever or to perform balloon expansion or stenting in a patient meet the following criteria: (1) Residual stenosis more than 70% or (2) impairment of distal blood flow, or (3) evidence of recanalized artery re-occlusion.

## Materials and Methods

### Ethics Statement and Participants

EAST study was approved by the Institutional Review Board at the Beijing Tiantan Hospital and other centers. All the patients gave written informed consent to participate and the privacy of patients was strictly protected (16). Clinical trial registration NO. NCT02350283. This multicenter prospective study enrolled 149 acute large arterial occlusion patients of anterior circulation who had undergone endovascular treatment with stent retrievers in 17 study centers in China between January 2015 and August 2015. Eligible patients were more than 18 years old, diagnosed of ischemic stroke, had stroke symptoms present for at least 30 min and had not significantly improved before treatment. Patients had no pre-stroke functional dependence (pre-stroke mRS ≤ 1), had a NIHSS ≥ 8 and < 30 and was able to be treated within 12 h of stroke symptoms onset with minimum of one deployment of the Solitaire Device (Onset time is defined as the last time when the patient was witnessed to be at baseline). Patients were confirmed to have symptomatic intracranial occlusion, based on CTA/MRA or DSA, at one or more of the following locations: Carotid T/L, M1 MCA, or M2-MCA equivalent affecting at least 50% of MCA territory. Patients with a baseline non-contrast CT (NCCT) or DWI revealed moderate/large ischemic core which defined as extensive early ischemic changes of Alberta Stroke Program Early CT Score (ASPECTS) < 7 in the area of LVO or DWI lesion volume more than 50 ml were excluded from our study.

For this study, 3 months' functional independence (modified Rankin scale score 0–2 vs. 3–6) was the main outcome analyzed. Arterial recanalization of the occluded artery was measured by modified Thrombolysis in Cerebral Infarction (mTICI) score of 2b or 3 (17). Symptomatic intracranial hemorrhage (sICH) was defined as parenchymal, subarachnoid or intraventricular hemorrhage detected by CT or MRI, associated with neurologic worsening of at least 4 points on the NIHSS, according to the second European–Australasian Acute Stroke Study (ECASS II) criteria (18). Parenchymal hematoma type 2 (PH-2) was defined as blood clots in 30% of the infarcted area with a substantial space-occupying effect according to ECASS II study. Excellent outcome was defined as mRS 0–1.

The association of clinical outcomes and presenting glucose was evaluated in the following two methods: (1) continuous variable of presenting glucose level and (2) admission hyperglycemia state (glucose level > 7.8 mmol /L).

### Statistical Analyses

Based on variable characteristics, the percentage, median (inter- quartile range) or mean (±standard deviation) of clinical data is reported. For categorical variables, the χ^2^ test and Fisher exact test used to do appropriate analysis. For continuous variables, the in- dependent samples *t-*test or the Mann–Whitney *U*-test were used to do appropriate analysis. For evaluation of relationship of clinical outcomes and hyperglycemia (or glucose levels, which were showed as the change in outcome for per 1 mmol/L increase in glucose), multiple logistic regression analysis was used for analysis. The potential confounding variables included in this analysis were age, sex, height, smoking status, NIHSS score at baseline, occlusion location, TOAST type, IV tPA status, Glycoprotein (GP) 2b3a inhibitor use and recanalization status. We further evaluated the odds ratios (ORs) and 95% confidence intervals (CIs). Statistically significant was defined as *P* < 0.05.

## Results

This prospective study recruited 149 acute anterior circulation proximal arterial occlusion patients at 17 stroke centers across China. There were 57 female patients and 92 male patients. Patients' mean age was 62.4 ± 12.4 years. All the patients were followed-up at 90 days. The pre-thrombectomy median NIHSS scores was 16 (IQR: 12–20). The median ASPECTS was 9 (IQR: 9–10). The occlusion site confirmed by DSA was ICA (T/L) in 76 cases (51%), M1 in 48 cases (32.2%), and M2 in 25 cases (16.8%). The median time from symptom onset to admission to the emergency room was 132 min (IQR: 75–210 min). Table 1 presented the summary of patients' clinical and treatment characteristics.

**Table 1:** Baseline and treatment characteristics of AIS patients receiving MT.

**Characteristic**	**Value** **(*n* = 149)**
**DEMOGRAPHIC CHARACTERISTICS**	
Age, median (SD)—yr	62.4 (12.4)
Female, no. (%)	57 (38.3)
**Medical history, *n* (%)**	
Hypertension	84 (56.4)
Diabetes mellitus	14 (9.4)
AF	60 (40.3)
**CLINICAL CHARACTERISTICS**	
NIHSS score, median (IQR)	16 (12–20)
Systolic BP at arrival, median (IQR)—mm Hg	141 (126–165)
ASPECTS on CT, median (IQR)	9 (9–10)
**Location of occlusion site, *n* (%)**	
ICA(T/L)	47 (31.5)
M1	78 (52.3)
Single M2	24 (16.1)
**Toast type, *n* (%)**	
LAA	73 (49.0)
CE	66 (44.3)
SOE	10 (6.7)
**ANESTHESIA TYPE, *N* (%)**	
General anesthesia	44 (29.5)
Conscious sedation	105 (70.5)
**THROMBECTOMY TREATMENT**	
Pre-thrombectomy IV alteplase, *n* (%)	25 (16.8)
Stenosis of occlusion artery, *n* (%)	47 (31.5)
Tandem lesion, *n* (%)	29 (19.5)
Acute ipsilateral carotid angioplasty, *n* (%)	16 (10.7)
Retrieval times, median (IQR)	2 (1–3)
GP IIb/IIIa inhibitor given, *n* (%)	45 (30.2)
**WORKFLOW TIME, MEDIAN (IQR)—MIN**	
Onset-to-Door time	132 (75–210)
Door-to-Puncture time	110 (67–160)
Puncture-to-Recanalization time	60 (38–94)
Onset-to-Recanalization time	308 (240–451)
mTICI 2b-3, *n* (%)	141 (94.6)
Symptomatic ICH, *n* (%)	6 (4.0)
Post-thrombectomy 24 h NIHSS score, median (IQR)	9 (4–15)
**FOLLOW UP RESULTS**	
mRS at 90 days, *n* (%)
0	29 (19.5)
1	35 (23.5)
2	19 (12.8)
3	16 (10.7)
4	25 (16.8)
5	7 (4.7)
6	18 (12.1)
mRS 0–2 at 90 days, *n* (%)	83 (55.7)

*SD, standard deviation; BP, blood pressure; ICA, internal carotid artery; M1-MCA, M1 segment of middle cerebral artery; TOAST, the trial of Org 10172 in acute stroke treatment; LAA, large artery atherosclerosis; CE, cardioembolism; SOE, stroke of other determined etiology; SUE, stroke of other undetermined etiology; IQR, inter quartile range; NCCT, non-contrast CT; IQR, inter quartile range; IV, intravenous; GP, glycoprotein; mTICI, modified thrombolysis in cerebral infarction; ICH, intracranial hemorrhage; mRS, modified Rankin Scale*.

After thrombectomy, 141 patients (94.6%) achieved successful recanalization (mTICI 2b/3). The time between admission and groin puncture (Door-to-Puncture time) was 110 min (IQR: 67–160 min), and the median time from groin puncture to first recanalization (Puncture-to-Recanalization time) was 60 min (IQR: 38–94 min). The median time from stroke onset to first recanalization (Onset-to-Recanalization time) was 308 min (IQR: 240–451 min). During thrombectomy, occluded artery atherosclerotic stenosis was found in 48 patients (32.2%) and 34 of them (22.8%) were treated with angioplasty. Tandem lesions were found in 29 patients (19.5%) and ipsilateral carotid angioplasty was performed in 16 patients (10.7%).

All the patients were followed up at 90 days after enrollment. Eighty-three patients got functional independence (mRS 0–2). This represented 55.7% of all patients and 63.4% of the survivors. In the perioperative period, 6 (4%) cases occurred sICH (4%) after mechanical thrombectomy. The mortality rate was 12.1% on 90-days follow-up (Table 1).

Patients' general characteristics according to whether hyperglycemia or not at presentation were showed in Table 2. The levels of glucose were higher in female patients (*P* = 0.014) and correlated with shorter in height (*P* = 0.046). The mean glucose levels at admission did not differ significantly between complete reperfusion (mTICI 2b-3) patients and patients with incomplete reperfusion (7.17 vs. 8.58 mg/dL; *P* = 0.085).

**Table 2:** Baseline and treatment characteristics for patients with different admission hyperglycemia state.

	**Glucose** **< 7.8 mmol/L**	**Glucose** **> = 7.8 mmol/L**	***P*-value**
Age, mean (SD)	63 (13.04)	65.44 (9.52)	0.133
Male, %	76/113 (67.3%)	16/36 (44.4%)	0.014
AF, %	46/113 (40.7%)	14/16 (38.9%)	0.846
DM, %	5/113 (4.4%)	9/36 (25%)	0.001
Hypertension, %	60/113 (53.1%)	24/36 (66.7%)	0.153
Height, mean (SD)	166 (7.15)	163.83 (7.58)	0.046
Weight, mean (SD)	66.18 (9.98)	64.07 (9.48)	0.242
SBP, mean (SD)	140.00 (25.40)	147.67 (27.92)	0.436
DBP, mean (SD)	83.88 (14.831)	86.53 (19.08)	0.515
NIHSS, median (IQR)	16 (12–19)	17.5 (13–21)	0.180
Smoking status, %			0.161
NO	59/113 (52.2%)	25/36 (69.4%)	
Smoking	38/113 (32.7%)	5/36 (13.9%)	
Cessation	17/113 (15.0%)	6/36 (16.7%)	
Pre-mRS, %			0.680
1	7/113 (6.2%)	1/36 (2.8%)	
0	107/113 (93.8%)	28/36 (77.8%)	
IV rtPA, %	17/113 (15.0%)	8/36 (22.2%)	0.316
ASPECTS, %	9 (8–10)	9 (8–10)	0.173
7	11/113 (9.7%)	7/36 (19.4%)	
8	21/113 (18.6%)	4/36 (11.1%)	
9	33/113 (29.2%)	15/36 (41.7%)	
10	48/113 (42.5%)	10/36 (27.8%)	
General anesthesia, %	36/113 (31.9%)	8/36 (22.2%)	0.270
Location, %			0.561
ICA	36/113 (31.9%)	11/36 (30.6%)	
M1	59/113 (52.2%)	19/36 (52.8%)	
M2	18/113 (15.9%)	6/36 (16.7%)	
Onset-to-Door time, median (IQR)—min	128 (63–215)	156 (93–197)	0.493
Door-to-Puncture time, median (IQR)—min	110 (60–160)	112 (75–159)	0.899
Puncture-to-Recanalization time, median (IQR)—min	62 (38–94)	45 (37–93)	0.323
Onset-to-Recanalization time, median (IQR)—min	297 (228–458)	336 (254–419)	0.778

*SD, standard deviation; BP, blood pressure; ICA, internal carotid artery; M1-MCA, M1 segment of middle cerebral artery; TOAST, the trial of Org 10172 in acute stroke treatment; LAA, large artery atherosclerosis; CE, cardioembolism; SOE, stroke of other determined etiology; SUE, stroke of other undetermined etiology; IQR, inter quartile range; NCCT, non-contrast CT*.

Patients who exhibited a functional independence at 90 day's follow-up showed significantly lower levels of glucose than those who did not (6.49 vs. 8.19 mmol/L; *P* < 0.001). After adjusting for covariates, for every 1 mmol/L increase of glucose, the patients were 83% less likely to have a functional independence at 3 months (OR 0.17, 95% CI 0.06–0.45; *P* < 0.001).

Patients with excellent outcome also had a significant lower glucose level than those who did not (6.43 vs. 7.86 mmol/L; *P* < 0.001). This study also found that sICH was significantly correlated with glucose level. Patients with sICH had a significantly higher glucose level than those who did not (10.09 vs. 7.12 mmol/L; *P* = 0.032). After adjusting for covariates, for every 1 mmol/L increase in glucose, the patients were 8.2 times more likely to exhibit a sICH after thrombectomy (OR 8.2, 95% CI 1.13–59.57; *P* = 0.038). Regarding other outcomes, such as recanalization status, dramatic neurologic improvement at 24 h, death, all intracranial hemorrhage and PH-2 ICH, the mean levels of glucose were similar for patients who did and did not experience each clinical outcome (Table 3).

**Table 3:** Association of glucose levels at presentation with clinical outcomes by univariable and multivariable logistic regression.

	***n***	**Glucose, mmol/L** **(Mean ± SD)**	*P*-value^*^	**Unadjusted OR** **(95% CI)**	***P*-value**	**Adjusted OR** **(95% CI)**	***P*-value**
Recanalization			0.130	0.87 (0.71–1.05)	0.148	0.25 (0.05–1.21)	0.085
2b−3	141	7.17 ± 2.49					
0–2a	8	8.58 ± 3.65					
Dramatic neurologic improvement at 24 h			0.110	0.89 (0.77–1.03)	0.119	0.54 (0.22–1.33)	0.182
Yes	62	6.85 ± 1.95					
No	87	7.53 ± 2.91					
Functional independence			< 0.001	0.70 (0.57–0.85)	< 0.001	0.17 (0.06–0.45)	< 0.001
mRS 0–2	83	6.49 ± 1.45					
mRS 3–6	66	8.19 ± 3.27					
Excellent outcome			< 0.001	0.72 (0.58–0.89)	0.002	0.23 (0.08–0.64)	0.005
mRS 0–1	64	6.43 ± 1.60					
mRS 2–6	85	7.86 ± 2.97					
No disability with daily activities			< 0.001	0.74 (0.61–0.90)	0.003	0.30 (0.12–0.79)	0.014
BI score 95–100	70	6.51 ± 1.54					
BI score < 95	79	7.89 ± 3.09					
Death			0.084	1.23 (1.05–1.43)	0.009	2.13 (0.63–7.34)	0.224
Yes	18	7.02 ± 2.21					
No	131	8.90 ± 4.09					
sICH			0.032	1.27 (1.04–1.54)	0.018	8.20 (1.13–59.57)	0.038
Yes	6	10.09 ± 4.38					
No	143	7.12 ± 2.42					
All ICH			0.231	0.90 (0.079–1.04)	0.145	1.22 (0.45–3.28)	0.698
Yes	34	7.82 ± 3.08					
No	115	7.07 ± 2.39					
PH-2 ICH			0.079	1.212 (0.96–1.53)	0.106	2.561 (0.19–33.96)	0.476
Yes	4	9.48 ± 3.72					
No	145	7.18 ± 2.52					

*AF, atrial fibrillation; CI, confidence interval; ICH, intracerebral hemorrhage; mRS, modified Rankin Scale; mTICI, modified thrombolysis in cerebral infarction; NIHSS, National Institutes of Health Stroke Scale; OR, odds ratio; PH, parenchymal hematoma type 2; sICH, symptomatic intracranial hemorrhage*.

* *P-values for continuous variables were calculated using t-tests (when the mean is reported), or the Wilcoxon test (when the median is reported); P-values for discrete variables were calculated using Fisher exact test*.

*All values were adjusted with age, sex, height, smoking status, NIHSS score at baseline, occlusion location, TOAST type, IV tPA and recanalization status*.

*Dramatic neurologic improvement was defined as a reduction of at least Dr on the NIHSS or a score of 0 to 2 at 24 h*.

*Scores on the modified Rankin scale were analyzed for an outcome with functional independence (score of 0–2) or an excellent outcome (score of 0 or 1)*.

*Scores on the Barthel Index range from 0 to 100, with higher values indicating good performance of daily living activities. A score between 95 and 100 indicates no disability that interferes with daily activities*.

*Symptomatic intracranial hemorrhage was defined as neurologic deterioration (an increase of 4 or more points in the score on the NIHSS) and evidence of intracranial hemorrhage on imaging studies (ECASS II criteria)*.

*Odds ratio for experiencing the first listed outcome; The incremental unit of glucose is 1 mmol/L; for example, for every 1 mmol/L increase in the glucose level, the patients were ≈30% less likely to exhibit a good outcome (mRS score 0–2)*.

The correlation between hyperglycemia (glucose level ≥ 7.8 mmol/L) and radiological/clinical outcomes at 90 days' follow-up were presented in Table 4. Patients with hyperglycemia at presentation were less likely to have a functional independence at 90 days than those without hyperglycemia (22.2 vs. 66.4%, *P* = 0.01). In multivariable analysis, hyperglycemia was correlated with a poor outcome at 90 days (OR 0.75, 95% CI 0.61–0.92; *P* = 0.005) independently. Similarly, excellent outcome was significantly lower among patients who presented with hyperglycemia vs. normoglycemia (16.7 vs. 51.3%, *P* = 0.017). The rates of recanalization status, dramatic neurologic improvement at 24 h, death, PH-2, and any ICH did not have any difference for patients with and without hyperglycemia.

**Table 4:** Association of Hyperglycemia at presentation with clinical outcomes by univariable and multivariable logistic regression.

	**Glucose** **< 7.8 mmol/L**	**Glucose** **> = 7.8 mmol/L**	**Unadjusted OR** **(95% CI)**	***P*-value**	**Adjusted OR** **(95% CI)**	***P*-value**
Recanalization			0.19 (0.06–0.61)	0.183	0.88 (0.70–1.10)	0.262
mTICI 2b−3	109/113 (96.5%)	32/36 (88.9%)				
mTICI 0–2a	4/113 (3.5%)	4/36 (11.1%)				
Dramatic neurologic improvement at 24 h			0.45 (0.20–1.02)	0.053	0.93 (0.79–1.09)	0.376
Yes	52/113 (46.0%)	10/36 (27.8%)				
No	61/113 (54.0%)	26/36 (72.2%)				
Functional independence			0.15 (0.06–0.35)	< 0.001	0.75 (0.61–0.92)	0.005
mRS 0–2	75/113 (66.4%)	8/36 (22.2%)				
mRS 3–6	38/113 (33.6%)	28/36 (77.8%)				
Excellent outcome			0.19 (0.07–0.49)	< 0.001	0.77 (0.62–0.95)	0.017
mRS 0–1	58/113 (51.3%)	6/36 (16.7%)				
mRS 2–6	55/113 (48.7%)	30/36 (83.3%)				
No disability with daily activities			0.24 (0.1–0.56)	0.001	0.81 (0.66–0.98)	0.029
BI score 95–100	62/113 (54.9%)	8/36 (22.2%)				
BI score < 95	51/113 (45.1%)	28/36 (77.8%)				
Death			2.94 (1.06–8.16)	0.032	1.15 (0.95–1.38)	0.144
Yes	10/113 (8.8%)	8/36 (22.2%)				
No	104/113 (91.2%)	28/36 (77.8%)				
sICH			6.94 (1.22–39.61)	0.013	1.25 (0.97–1.61)	0.090
Yes	2/113 (1.8%)	4/36 (11.1%)				
No	111/113 (98.2%)	32/36 (88.9%)				
All ICH			0.85 (0.36–2.05)	0.720	0.92 (0.79–1.08)	0.301
Yes	25/113 (22.1%)	9/36 (25%)				
No	89/113 (77.9%)	27/36 (75%)				
PH-2 ICH			10.18 (1.03–101.17)	0.044	–	–
Yes	1/113 (0.9%)	3/36 (8.3%)				
No	112/113 (99.1%)	33/36 (91.7%)				

*AF, atrial fibrillation; CI, confidence interval; ICH, intracerebral hemorrhage; mRS, modified Rankin Scale; mTICI, modified thrombolysis in cerebral infarction; NIHSS, National Institutes of Health Stroke Scale; OR, odds ratio; PH, parenchymal hematoma type 2; sICH, symptomatic intracranial hemorrhage*.

* *P-values were calculated using Fisher exact test*.

*All values were adjusted with age, sex, height, smoking status, NIHSS score at baseline, occlusion location, TOAST type, IV tPA and recanalization status*.

*Dramatic neurologic improvement was defined as a reduction of at least Dr on the NIHSS or a score of 0 to 2 at 24 h*.

*Scores on the modified Rankin scale were analyzed for an outcome with functional independence (score of 0–2) or an excellent outcome (score of 0 or 1)*.

*Scores on the Barthel Index range from 0 to 100, with higher values indicating good performance of daily living activities. A score between 95 and 100 indicates no disability that interferes with daily activities*.

*Symptomatic intracranial hemorrhage was defined as neurologic deterioration (an increase of 4 or more points in the score on the NIHSS) and evidence of intracranial hemorrhage on imaging studies (ECASS II criteria)*.

*Odds ratio for experiencing the first listed outcome for patients with glucose level > = 7.8 mmol/L; for example, patients with a glucose level > = 7.8 mmol/L were ≈85% less likely to exhibit an excellent outcome (mRS score 0–1)*.

## Discussion

Our study demonstrated that in patients treated with Solitaire stent thrombectomy for anterior circulation LVO, higher serum glucose at admission, and hyperglycemia were correlated with lower odds of functional independence (defined as mRS scores of 0–2) at 90 days' follow-up independently. These association has statistical significance after adjusted by age, sex, height, smoking status, NIHSS score at baseline, occlusion location, TOAST type, IV tPA, and recanalization status after thrombectomy. Our study also found higher admission serum glucose was also associated with sICH after thrombectomy. The influence of hyperglycemia was similar for patients without diabetes in our study, but showed no difference for patients with diabetes because of limited case number (Supplement Tables 1, 2). In our study the patients under thrombectomy is different in population compared to previous Western studies. ICAS was common in our study and rescue therapy with angioplasty or stenting during thrombectomy is allowed in our study.

Reported studies showed the correlation of hyperglycemia at admission with adverse outcomes in AIS patients treated through IVT. In addition, admission hyperglycemia was associated with a higher risk of sICH in different risk prediction scores (19, 20) and lower odds of reperfusion induced by tPA (3) for patients under the treatment of thrombolysis. The Increasing of glucose levels at admission also associated with more extensive infarction areas in the final (21, 22). Besides these, in different cohorts of AIS patients treated under IVT, hyperglycemia at presentation was proved to be an independent predictor of 90 days' poor functional outcome and early mortality (23).

When compared with fibrinolytics alone, MT for AIS didn't increase sICH or mortality (8, 9, 11). Previous study had shown that for IV alteplase thrombolysis, hyperglycemia was correlated with a decrease of artery recanalization (24). The mechanisms for this relationship maybe that hyperglycemia was correlated with atherosclerotic disease or was related to antifibrinolytic effect (25). However, our study showed that hyperglycemia was not associated with the state of reperfusion after MT.

Our study's result was in accordance with the study showing the lack of an correlation between artery recanalization and diabetes mellitus or glucose levels in patients under different kinds of intra-arterial therapy (26). This study found that for hyperglycemic patients MT could overcome the potentially reduced fibrinolytic capacity. MT increased recanalization rate and brought more ischemia and reperfusion injury after reperfusion which allowed hyperglycemic to show the effect on reperfusion brain.

Our findings are consistent with the recently published MR CLEAN pre-trial registry (27). They found that for AIS after MT hyperglycemia in admission (glucose more than 7.8 mmol/L) and impaired fasting glucose (more than 5.5 mmol/L) in the first week after admission were correlated with unfavorable short term outcomes and a higher risk of sICH. However, they assessed functional outcomes on discharge and this study used 3 months' mRS to value functional outcomes.

Recently a retrospective study of previously prospectively collected database found that for LVO patients under MT treatment, hyperglycemia at admission was associated with a higher likelihood of mortality at 3 months and lower odds of functional improvement at 3 months in terms of shift in mRS score. However, they did not found hyperglycemia and serum glucose at admission associate with functional independence at 3 months in multivariable analyses. What's more, different from this study which the technique of A Direct Aspiration First Pass Technique (ADAPT) was used in 23% patients, all the patients in our study were treated with solitaire stent thrombectomy.

Another *post-hoc* analysis of SWIFT (Solitaire flow restoration device vs. the Merci Retriever in patients with AIS) trial showed that in patients treated with thrombectomy hyperglycemia was independently associated with 3 months' worse outcome and for incomplete reperfusion patients after MT hyperglycemia's negative effects may have been exacerbated. For complete reperfusion patients, there was no statistical significance for presenting glucose levels differences by final outcome. In this study, only 51% patients got reperfusion (defined by TIMI 3). However, our data showed a high reperfusion (defined by mTICI 2b-3) rate of 94.6% and hyperglycemia or serum glucose at admission was not correlated with the likelihood of recanalization status after thrombectomy.

Even though we found the relationship between hyperglycemia and poor outcome after thrombectomy, it is controversial whether hyperglycemia should be strictly managed in these patients. In a randomized controlled trial, when compared with control patients with vessel occlusion, greater infarct growth was found in patients treated with glucose–potassium–insulin (28). Another study revealed that for patients with good collaterals, higher glucose levels reduced the likelihood of good outcome, but for patients with poor collaterals this effects on the outcome are less significant (29). This study found that good collaterals at presentation may be targets for more intensive glucose control and future studies relating to glucose management.

This study had limitations. First, this was a *post-hoc* analysis of a prospective registry study. Second, the effect of in-hospital management of hyperglycemia was not investigated. Third, moderate sample size limited study power. We found significant statistical significance (*p* = 0.009) in the correlation between admission hyperglycemia and mortality on the univariable analyses but didn't find statistical significance on multivariable analyses may be attributed to the moderate sample (*n* = 149) of our study population.

## Conclusions

Our study revealed that for patients treated with Solitaire stent thrombectomy of anterior circulation LVO, higher admission serum glucose, and hyperglycemia were independently associated with lower functional independence at 3 months. Higher admission serum glucose was also associated with sICH after thrombectomy. These findings suggest that high glucose level at admission should be carefully concerned for AIS patients under thrombectomy with solitaire stent. Further prospective investigation of the intervention of admission hyperglycemia should be suggested.

## Author Contributions

ZM, YiW, and YoW conceived and led the project. XL, CW, XS, and LS performed the laboratory examination. FG, NM, DM, LipL, and XZ supervised and performed quality control for the study. BJ, LiaL, BW, and YP acquired the data (acquired and managed patients, selected, and characterized samples). XH and RL co-wrote the manuscript with input from all co-authors.

### Conflict of Interest Statement

The authors declare that the research was conducted in the absence of any commercial or financial relationships that could be construed as a potential conflict of interest.
