# Supplementary material for: Effect of Hyperglycemia at Presentation on Outcomes in Acute Large Artery Occlusion Patients Treated With Solitaire Stent Thrombectomy
Source: Front Neurol. 2019 Feb 19;10:71. doi: 10.3389/fneur.2019.00071 (PMC6390827; doi:10.3389/fneur.2019.00071)
Supplement: Supplementary file 1 [file Table_1.DOCX]

**Supplement Table 1. Association of Hyperglycemia at presentation with clinical outcomes for patients without diabetes (n=135)**

|  | **Glucose**  **<7.8mmol/L** | **Glucose**  **>=7.8mmol/L** | **Unadjusted OR**  **(95% CI)** | **P value** |
| --- | --- | --- | --- | --- |
| Functional independence |  |  | 0.15(0.06-0.32) | <0.001 |
| mRS 0-2 | 71/108(65.7%) | 6/27(22.2%) |  |  |
| mRS 3-6 | 37/108(34.3%) | 21/27(77.8%) |  |  |
| Excellent outcome |  |  | 0.22(0.08-0.62) | 0.002 |
| mRS 0-1 | 55/108(50.9%) | 5/27(18.5%) |  |  |
| mRS 2-6 | 53/108(49.1%) | 22/27(81.5%) |  |  |
| Death |  |  | 1.91(0.54-6.76) | 0.307* |
| yes | 9/108(8.3%) | 4/27(14.8%) |  |  |
| no | 99/108(91.7%) | 23/27(85.2%) |  |  |
| sICH |  |  | 6.63(1.05-41.85) | 0.054 |
| yes | 2/108(1.9%) | 3/27(11.1%) |  |  |
| no | 106/108(98.1%) | 24/27(88.9%) |  |  |

**Abbreviations:** AF=atrial fibrillation; CI=confidence interval; ICH=intracerebral haemorrhage; mRS=modified Rankin Scale; mTICI=modified Thrombolysis in Cerebral Infarction; NIHSS=National Institutes of Health Stroke Scale; OR=odds ratio; PH=parenchymal hematoma type 2; sICH= symptomatic intracranial haemorrhage;

*P values were calculated using Fisher exact test.
