# Supplementary material for: Effect of Hyperglycemia at Presentation on Outcomes in Acute Large Artery Occlusion Patients Treated With Solitaire Stent Thrombectomy
Source: Front Neurol. 2019 Feb 19;10:71. doi: 10.3389/fneur.2019.00071 (PMC6390827; doi:10.3389/fneur.2019.00071)
Supplement: Supplementary file 2 [file Table_2.DOCX]

**Supplement Table 2. Association of Hyperglycemia at presentation with clinical outcomes for patients with diabetes (n=14)**

|  | **Glucose**  **<7.8mmol/L** | **Glucose**  **>=7.8mmol/L** | **Unadjusted OR**  **(95% CI)** | **P value*** |
| --- | --- | --- | --- | --- |
| Functional independence |  |  | 0.07(0.01-1.06) | 0.091 |
| mRS 0-2 | 4/5(80.0%) | 2/9(22.2%) |  |  |
| mRS 3-6 | 1/5(20.0%) | 7/9(77.8%) |  |  |
| Excellent outcome |  |  | 0.08(0.05-1.29) | 0.095 |
| mRS 0-1 | 3/5(60.0%) | 1/9(11.1%) |  |  |
| mRS 2-6 | 2/5(40.0%) | 8/9(88.9%) |  |  |
| Death |  |  | 1.91(0.54-6.76) | 0.307 |
| yes | 1/5(20.0%) | 4/9(44.4%) |  |  |
| no | 4/5(80.0%) | 5/9(55.6%) |  |  |
| sICH |  |  | 1.13(0.89-1.42) | 1.000 |
| yes | 0/5(0.0%) | 1/9(11.1%) |  |  |
| no | 5/5(100.0%) | 8/9(88.9%) |  |  |

**Abbreviations:** AF=atrial fibrillation; CI=confidence interval; ICH=intracerebral haemorrhage; mRS=modified Rankin Scale; mTICI=modified Thrombolysis in Cerebral Infarction; NIHSS=National Institutes of Health Stroke Scale; OR=odds ratio; PH=parenchymal hematoma type 2; sICH= symptomatic intracranial haemorrhage;

*P values were calculated using Fisher exact test.
